# Supplementary material for: Getting More Out of Biomedical Documents with GATE's Full Lifecycle Open Source Text Analytics
Source: PLoS Comput Biol. 2013 Feb 7;9(2):e1002854. doi: 10.1371/journal.pcbi.1002854 (PMC3567135; doi:10.1371/journal.pcbi.1002854)
Supplement: Dataset S1 — GATE software. Dataset S1 bundled with this paper contains a distribution of GATE (or see http://gate.ac.uk/download/). (TGZ) [file pcbi.1002854.s001.tgz › plos-gate/plugins/Web_Crawler_Websphinx/doc/Crawler.doc]

**A crawler plugin for GATE**

1. **Introduction**

The crawler Plugin is a way to enable GATE to be used for a corpus that is built using a web crawl. The crawler itself is Websphinx [http://www.cs.cmu.edu/~rcm/**websphinx**/](http://www.cs.cmu.edu/~rcm/websphinx/) This is a JAVA based multithreaded web crawler that can be customized for any application. In order to use this plugin it may be required that the websphinx.jar file be added in the required libraries in JBuilder.

The following document provides an overview of the Crawler PR.

The basic idea was to be able to specify a source URL and a depth to build the initial corpus upon which further processing could be done. The PR itself provides a number of helpful features to set various parameters of the crawl.

2. **Using the Crawler PR**

In order to use the processing resource you first need to load the plugin using the plugin manager.

**Figure 1: Load the Crawl PR from the plugin manager**

**Figure 2 Initialize the crawl PR**

Once the crawl PR is initialized the PR automatically creates a corpus named ‘crawl’ where all the documents from the web crawl would be stored. In order to use the crawler create a simple pipeline and add the crawl PR to the pipeline.

**Figure 3 Create the pipeline with a crawl PR in it.**

Once the crawl PR is created there will be a number of parameters that can be set based on the PR required. These are shown in the next diagram and are discussed below.

**Figure 4 The basic pipeline with the crawl PR in it**

The following describe the various options

1. depth : the depth to which the crawl should proceed.
2. dfs / bfs : dfs if true bfs if false
   a. Dfs : the crawler uses the depth first strategy for the crawl.
      i. Visits the nodes in dfs order until the specified depth limit is reached.
   b. Bfs : the crawler used the breadth first strategy for the crawl.
      i. Visits the nodes on bfs order until the specified depth limit is reached.
3. domain
   a. SUBTREE

Crawler visits only the descendents of the page specified as the root for the crawl.

b. WEB

Crawler visits all the pages on the web.

c. SERVER

Crawler visits only the pages that are present on the server where the root page is located.

4. max number of pages to be fetched
5. root the starting URL to be used for the crawl to begin
6. source is the corpus to be used that contains the documents from which the crawl must begin

Source is useful when the documents are fetched first from the google PR and then need to be crawled to expand the web graph further. At any time either the source or the root needs to be set.

Once the parameters are set, the crawl can be run and the documents fetched are added to the corpus crawl. The final figure shows the docuemts added to the corpus.

**Figure 5 the crawled pages are added to the crawl corpus**
